# Prevalence, Reasons, and Predisposing Factors Associated with 30-day Hospital Readmissions in Poland

**DOI:** 10.3390/ijerph16132339

**Published:** 2019-07-02

**Authors:** Jacek Kryś, Błażej Łyszczarz, Zofia Wyszkowska, Kornelia Kędziora-Kornatowska

**Affiliations:** 1 Antoni Jurasz University Hospital No. 1, 85-094 Bydgoszcz, Poland; 2 Department of Public Health, Faculty of Health Sciences, Nicolaus Copernicus University in Toruń, 85-830 Bydgoszcz, Poland; 3 Department of Organization and Management, Faculty of Management, University of Science and Technology, 85-790 Bydgoszcz, Poland; 4 Department and Clinic of Geriatrics, Faculty of Health Sciences, Nicolaus Copernicus University in Toruń, 85-094 Bydgoszcz, Poland

**Keywords:** patient readmission, hospitals, quality of healthcare, multilevel logistic regression

## Abstract

There is a growing interest in quality issues associated with hospital care, with readmissions (rehospitalizations) being one of the main areas of interest. Retrospective data from a 914-bed university hospital in Bydgoszcz, Poland, was used to identify 30-day readmissions in 2015. We developed a catalogue of reasons for rehospitalization and differentiated between planned and unplanned readmissions, as well as those related and unrelated to index (initial) hospitalization. Multilevel logistic regression was used to determine factors associated with readmission risk. A total of 12.5% of patients were readmitted within 30 days of being discharged. The highest readmission rates were identified in pediatric, transplantation, and urology patients. The highest share of readmissions was due to the specific nature of a disease and its routine treatment practice. Almost two-thirds of readmission cases were classified as unplanned and related to the index hospitalization. The following characteristics were associated with a higher risk of rehospitalization: female gender, residing >35 km from the hospital, longer than average and very short stays at index admission, higher comorbidity score, and admission to a high-volume hospital sector. Due to the importance of quality issues in health policy, the topic should be further pursued to identify evidence-based practices that would improve hospitals’ performance.

## 1. Introduction

Quality issues are a rapidly growing concern in the delivery of hospital services. Hospital financing constitutes the greatest share of health spending, making both policymakers and third-party payers (health insurers) more aware of the efficiency and quality of care delivered in inpatient settings [1,2,3,4]. In this context, a number of outcome-based quality measures have been proposed to assess the value for money in hospital care. Indicators quantifying hospital readmissions (rehospitalizations) are attracting the growing attention of researchers and stakeholders in the health sector [5,6,7]. Research on readmissions focuses on two main areas, namely the prediction of readmission risk associated with a patient’s characteristics [7,8] and the assessment of the preventability of readmission [9,10]. Considering the relevance of the problem for health policy practice, we observe a growing interest in linking the prevalence of readmission with provider reimbursement schemes. For example, US hospitals are subject to a maximum 3% reduction in Medicare refund if their rehospitalization rates are too high [11,12]. In Poland, the National Health Fund (Narodowy Fundusz Zdrowia)—the monopolistic public health insurer—does not reimburse rehospitalizations that occur within 14 days of discharge. Taking into consideration the organizational issues associated with the operation of hospitals, it has been documented that crowding in emergency departments (ED) has an impact on readmission rates in hospital wards [13,14,15]. Crowded EDs can negatively affect the quality of care due to long waiting times and delayed treatment. Furthermore, efforts to avoid crowding in EDs may require hospitals to accelerate discharges from inpatient clinics, in order to admit patients transferred from emergency settings. This, in turn, can lead to an increased risk of adverse outcomes, including readmissions [16].

With the growing availability of administrative data on hospital care, policymakers and providers are now able to make more informed choices regarding the efficiency and quality of care. However, despite the growing evidence on the predisposing factors and reasons for readmission, empirical findings are inconclusive. For example, a review on risk prediction models for hospital readmissions concludes that models designed for either comparative or clinical purposes perform poorly [6]. In addition, classifying and identifying those readmissions, which can be prevented, is a complex issue that requires further inspection. Moreover, the vast majority of evidence on readmissions comes from the US health system, which is characterized by unique organizational and financial settings. Only a few studies have used data from Europe [8,9,17,18] and, to date, no study on rehospitalizations in Poland has been published. For these reasons, and regarding the anticipated quality-enhancing reforms in the reimbursement system in Poland, we analyzed data on hospital readmissions from 2015 using medical records from a single university hospital in the city of Bydgoszcz. The aims of this study were to investigate the prevalence and structure of readmissions in the hospital’s operation, to examine the reasons for rehospitalizations, and to scrutinize patient characteristics that were associated with readmission risk.

## 2. Materials and Methods

In this study, we used data from a 914-bed university hospital (Antoni Jurasz University Hospital No. 1) in Bydgoszcz, Poland, which is the largest hospital in the northern region of kujawsko-pomorskie (region population in 2015: 2.08 million) in terms of employment. There is another university hospital in the same city, and another of similar size within a distance of 50 km. However, Jurasz Hospital is the only one in the region with the highest referral rate in all medical specialties. The choice of this entity was based on the fact that the hospital collects comparative data of satisfactory quality, which could be used in the study of readmissions. The data used to identify readmissions covered index (initial) hospitalizations from 1 January 2015 until 31 December 2015. However, because we used a 30-day period for defining readmission, we also examined hospitalizations from January 2016 to identify readmissions for which the index hospitalization took place in December 2015.

We used electronic medical records to identify hospitalizations and readmissions in all hospital wards which discharge patients from the hospital. With this approach, all wards providing intensive care were excluded.

A 30-day period was used to identify readmissions and the readmission rate (RaR) index was defined as follows:

RaR = (Readmissions*_n_* + Readmissions_*n*+30_)/(Hospitalizations*_n_* − Readmissions*_n_*)

where ‘*n*’ denotes the year 2015 and ‘*n* + 30’ refers to 30 days of the year 2016 which is the period when readmissions for hospitalizations from the last month of 2015 took place. A 30-day RaR is a common measure of readmission prevalence [5,6] and is extensively used in health services research concerning hospitalization processes [19,20,21,22]. We distinguished between two types of index hospitalizations, namely single- and multiple-stay. The former means that a patient was treated in a single clinic, while the latter refers to a hospitalization with multidisciplinary treatment in more than one clinic. In both cases, intensive care and emergency services were excluded. For a single-stay hospitalization, a readmission was defined as hospitalization to the same clinic or to another clinic that performs a similar range of medical procedures. With this approach, we clustered the clinics performing similar procedures into sectors. All the sectors, except pediatrics and internal medicine, include a single clinic (the pediatrics sector consists of departments engaged in surgery, allergology, gastroenterology, and hematology of pediatric patients; the internal medicine sector consist of cardiology, nephrology, hypertension, geriatrics, endocrinology, diabetology, and internal medicine clinics). For a multiple-stay hospitalization, admission to any clinic was considered to be a readmission. Distinguishing between single- and multiple-stay index admissions made it possible to account for the more complicated treatment of patients admitted to multiple clinics during one stay.

In order to identify the most plausible reason for a particular rehospitalization, each of the readmissions was subject to an evaluation by the physician who treated the patient during the rehospitalization. For that purpose, a set of reasons for rehospitalization based on the literature review [5,6,10] and on the opinion of hospital physicians was constructed with 14 alternative reasons. Some of these were further differentiated to reflect all possible reasons for readmissions (for the sake of brevity we do not show these reasons here; they are shown in Table 3 in the ‘Results’ section). To categorize the readmissions identified according to their relationship with the index hospitalization and whether a readmission was planned or unplanned, we introduced four categories of rehospitalizations: (A) planned and related to the index admission; (B) planned and unrelated to the index admission; (C) unplanned and related to the index admission; and (D) unplanned and unrelated to the index admission [23]. Each of the 14 reasons for readmission was assigned to one of the four groups. Because this research uses data from a single hospital, we were not able to identify cases in which patients discharged from this hospital were readmitted to other inpatient providers.

The analysis is limited to 2015 solely because of the complexity of the study design, which required the contribution of dozens of physicians to assess admission reasons. Potentially, additional years could be included in the analysis. However, it would be more time consuming for numerous hospital workers. Furthermore, the numbers of hospitalizations (34,008) and readmissions (3789) in a single year in our study are notably higher than in other recent studies which used data from a single hospital (see the second paragraph of the ‘Discussion’ for comparison). Moreover, taking into account the formal requirements of the statistical methods used, the number of observations from a single year appears to be sufficient.

### 2.1. Statistical Analysis

For univariate analysis, we used a chi-square test. For the assessment of the factors associated with readmission, we used multiple multilevel logistic regression with an odds ratio (OR) and 95% confidence intervals (CI) estimated. Statistical significance was determined by a *p*-value lower than 0.05 and two-sided tests were used.

We used multilevel logistic regression [24,25,26] with sector-specific random-effects and decided not to cluster admissions at a patient level. With this approach, we followed contemporary literature, which uses multilevel modeling without clustering hospitalizations at a patient level [27,28] because the number of readmissions per patient is too low to affect the estimates [22]. In our study, there was a mean rate of 1.4 hospitalizations per patient admitted, making the number of individual cases in each cluster (patient) too low for the within-patient effects to be notable. Stata (ver. 14.2, StataCorp LLC, College Station, TX, USA) command xtmelogit was used to estimate the regression model.

The outcome of interest in the multilevel logistic regression was 30-day all-cause readmission, which was defined in the manner explained above. The choice of potential explanatory variables was based on the literature review and the patient-level (level 1) covariates included:

- Patient’s gender (male or female);
- Patient’s age grouped into five categories (<5; 5–17; 18–29; 30–49; 50–65; >65 years);
- Place of residence grouped into six categories (single rural and five urban sub-categories depending on the population size: ≤20.000; 20.001–50.000; 50.001–200.000; 200.001–500.000; >500.000 population);
- Patient’s distance from home to hospital (≤15 km; 16–35 km; >35 km);
- Type of admission (emergency or scheduled);
- Day of admission (weekday or weekend);
- Day of discharge (weekday or weekend);
- Time of admission (7:00–14:59; 15:00–22:59; 23:00–6:59);
- Length of stay (LOS) categorized in intervals (≤1; 1.01–4.00; 4.01–7.00; 7.01–14.00; >14 days) [27];
- Age-adjusted Charlson comorbidity index [29,30] (ACCI) (0–1; 2–3; 4–5; ≥6 points [31,32]).

Moreover, three potential sector-level (level 2) variables were included in the analysis and these were the numbers of hospitalizations, physicians, and beds in particular sectors.

Although age, ACCI, and LOS are continuous variables, we categorized them into intervals as the association between these covariates and readmission is likely to be non-linear. We also tested the specifications with these variables being treated as continuous. However, these models proved to be worse in terms of statistical model characteristics.

The use of gender and age variables is a routine practice in patient outcome studies. The place of residence was a proxy for access to health care facilities as it is well recognized that health care accessibility is higher in (larger) cities [33,34], and this could have an effect on the rehospitalization risk. The higher comorbidity burden potentially elevates the risk of being readmitted. On the other hand, the effect of LOS during the index hospitalization is unpredictable and potentially is not linearly associated with readmission risk. An in-patient stay that is either too long or too short may result in a higher risk of rehospitalization. The distance from a patient’s home to the hospital can also be associated with the chance of being readmitted. Moreover, the day of the week of both admission and discharge as well as time of admission potentially affect outcomes associated with hospitalization [9,35]—both these factors were included in our analysis. Level 2 variables were included to control for scale effects in clinics’ operation.

The starting point for building the regression model was to include all the potential covariates (see the previous paragraphs) into a random intercept model. Backward elimination was applied to this model with a one-by-one exclusion of independent variables with *p* > 0.05, and the order of exclusion was determined by the decreasing *p*-value. The Akaike information criterion (AIC) and Bayesian information criterion (BIC) were used for selecting the model with a set of covariates that minimized AIC and BIC. If AIC and BIC favored different models, a specification with more covariates was chosen, provided that the dependent variable under consideration had a *p*-value < 0.1. Similar to other studies using multilevel methodology in readmission analyses, we did not use a random coefficient model [21,22,27,28,36] as we did not expect covariates’ coefficients to differ across sectors.

### 2.2. Ethics

The study was prospectively approved by the Bioethics Committee at Collegium Medicum, Nicolaus Copernicus University in Toruń (decision no. KB 25/2016). Only information that was routinely collected during hospitalization was used: demographics, diagnoses, procedures, the number of comorbidities, and lengths of stay of all inpatients from 1 January 2015 until 31 January 2016. The data were retrieved from patients’ electronic records by one of the investigators (JK) and a database was created in a spreadsheet. Due to the fact that we used anonymized electronic medical records, we did not seek written consent from the participants. The complete dataset used for the analysis is available as a Supplementary Materials (see S1 dataset for details).

## 3. Results

Among 34,008 hospitalizations in 2015, 3789 cases were readmitted within 30 days from the patient’s discharge from hospital, resulting in the RaR of 12.5%. The highest number of readmissions was observed among the eldest group (65+ years: 919 cases). However, RaR was highest among children (30.8% in the 0–4 years group and 28.6% in the 5–17 years group). Rehospitalizations were most frequent among those living in rural areas (RaR = 15.6%), and the value of RaR was highest for those living >35 km away from the hospital (RaR = 16.1%). Readmissions were observed more often in scheduled hospitalizations than in emergency admissions (13.3% vs. 11.4%; *p* < 0.01). Patients whose hospitalization began during weekdays were readmitted more often than those admitted on weekends (12.8% vs. 10.9%; *p* < 0.01). Similarly, the admissions with discharge on weekday were associated with higher RaR than hospitalizations with patient discharged on weekend (12.7% vs. 11.0%; *p* = 0.01). The highest rate was observed in those patients with an AACI score of 2–3 (RaR = 20.0%). Concerning hospital sectors’ characteristics, the highest RaR values were identified in those sectors with the greatest numbers of beds, physicians, and hospitalizations (Table 1).

Readmissions were most frequent in the pediatrics sector both in terms of the total number (1771) and RaR (33.9%). The rate was higher than 10% also in transplantation, urology, and vascular surgery sectors, with the values of 24.1%, 17.1%, and 11.8%, respectively. On the other hand, a less than 5% rate was identified in ten sectors, with no readmissions in rehabilitation and orthopedic rehabilitation (Table 2).

Regarding the reasons for readmission, the most frequently chosen was ‘specific nature of the disease and its routine treatment practice’ (code 8) with a 43% share of all rehospitalizations. Other frequent readmission reasons were: (14) radiotherapy sessions and chemotherapy cycles (14.0%); (9b) emergency rehospitalization due to disease different to that of the index hospitalization (6.3%); and (10) disease progression (5.8%). On the other hand, only a few readmissions were explained as possibly resulting from (13) ailments not confirmed during readmission, (7a and 7b) non-optimal therapy, and (5c) incomplete diagnostics during the index hospitalization (each reason—0.1%); as well as by early discharge of the patients due to various reasons including the unavailability of beds (4b—0.2%); the request of the patient (4d—0.3%); inadequate clinical assessment on discharge (4c—0.4%); and organizational factors (4a—1%) (Table 3).

More than 90% of the readmissions were related to the index hospitalization with the unplanned ones (category C) constituting 63.9% and the planned ones (category A) constituting 26.5% of all rehospitalizations. Most readmissions that were unrelated to the index hospitalization were unplanned (category D—6.4%) and the planned ones were identified as the rarest (category B—3.2%) (see Table 3 footnote).

The final specification of multilevel multiple logistic regression contains patient-level covariates of gender, age, distance from the patient’s home to hospital, LOS, ACCI, and the sector-level variable of the yearly number of hospitalizations (Table 4). The estimates show that the risk of readmission is slightly higher for women (odds ratio, OR = 1.14; 95% CI: 1.06–1.23). Compared to young adults aged 18–29, the risk of readmission is around four times lower for the two oldest groups. Concerning the distance from home, patients residing 36 or more kilometers from the hospital had somewhat higher odds of readmission (OR = 1.17; 95% CI: 1.08–1.27) compared to those living close to the hospital. Compared to admissions lasting 4.01 to 7 days (this category was chosen as reference because it contains the mean value of LOS), the shortest (<1 day) and longer (7.01 to 14 days and >14 days) hospitalizations had higher odds of readmission. Compared to a 0–1 ACCI score, higher comorbidity (score ≥ 2 in ACCI) was associated with 9.7–11.4 times higher odds of readmission. When it comes to sector-level variables, a higher number of hospitalizations in a sector was associated with a higher risk of readmission. The sectors with >3000 admissions yearly had 10-times higher odds than the ones with ≤1000 cases. However, the confidence intervals for this last variable were very wide. The discriminative power of the model was satisfactory, with a c-statistic value of 0.749, the model performed effectively in terms of correctly discriminating between high- and low-risk patients (Table 4).

## 4. Discussion

In this study, we analyzed the prevalence, reasons for, and predisposing factors of 30-day all-cause readmissions in a single university hospital in Poland. The number of readmissions was 3789 in 2015, translating to a RaR value of 12.5%.

The current study is the first to analyze rehospitalizations in Poland, therefore, the RaR of 12.5% can only be compared to results from other countries. For comparability of findings from different settings, we compare our results only to those studies using a 30-day period to identify all-cause readmissions. A study using data from a community non-teaching hospital in Italy reported RaR of 10.2% among 2252 patients (for the period 2005–2007). However, this result was obtained for adult patients only [9]. An American study based on data from a university hospital in California analyzed 10,359 admissions (2006–2008) discharged from general medicine service with RaR of 17.0% [37]. Another recent Italian study identified the rate of unplanned readmissions as 11.6% at Pisa University Hospital (data for 5388 admissions in 2012) [38]. A prospective cohort study from a single Belgian university hospital (data for years 2011–2012) reported a RaR of 18.6% for emergency department patients aged >75 years [39]. The above examples identified a high variation in the readmission rate in single-center settings, and the range of RaR was also wide when one considers studies using data on the general population. In Israel, 16.8% of adult admissions of those insured by Clatit (not-for-profit integrated health care organization covering 54% of the Israeli population) were readmitted within 30 days from the index hospitalization in the 1st quarter of 2010 [40]. With data from 70 acute-care American hospitals, a RaR of 11.9% was identified among ~1.2 million adults hospitalized from 2006 to 2008 [41]. A nation-wide American study using data from the Medicare fee-for-service program showed that 19.6% of almost 12 million patients hospitalized in the period 2003–2004 were readmitted within 30 days [42]. Comparing RaR values from the range of studies, it seems that methodological differences and various organizational settings of hospital care in particular countries do not allow for making direct comparisons between the readmission rate identified here and in other studies.

According to our estimates, almost half of all rehospitalizations were in the pediatrics sector, where the rate of readmission was highest (RaR = 33.4%). Almost 80% of all rehospitalizations were identified in four sectors; apart from pediatrics, these were: internal medicine (18.6% of total), urology (8.3%), and ophthalmology (7.8%). Considering the RaR values in particular sectors, pediatrics hospitalizations seem to be at the highest risk of readmission, followed by transplantation (RaR = 23.7%), and urology (RaR = 17%). Together, raw numbers and prevalence show that the magnitude of readmissions was most severe in pediatrics, urology, and internal medicine. On the other hand, in ten of the hospital sectors, RaR was <5% and readmissions accounted for 5.6% of all rehospitalization cases.

Regarding reasons for rehospitalization, physicians evaluating readmissions pointed to the specific nature of a disease and its routine treatment practice as the most common reason (code 8–43% of total cases). This can be explained in two ways. First, as it is the only hospital in the region with the highest referral rate in all medical specialties, it treats the most complicated and difficult cases which are more susceptible to readmission. Secondly, this response could have been chosen by medical staff for opportunistic reasons; regarding personal responsibility for treatment outcomes, selecting this readmission reason was the safest option. The second most prevalent reason for rehospitalization was radiotherapy sessions and chemotherapy cycles (code 14–13.9% of cases) provided in the pediatric sector, which are considered unavoidable because they result from a typical clinical pathway. Almost 10% of all cases were readmissions due to diseases other than that of the index hospitalization (codes 9a and 9b). A total of 72% of rehospitalizations due to this reason were identified among patients with >1 comorbidity and in 98% cases, the index admission and readmission took place in the same sector, possibly reflecting the fact that a large proportion of these readmissions were a continuation of treatment. Less than 1% of rehospitalizations were due to non-optimal performance of physicians during the index hospitalization (codes 4c, 5c, and 7b). A low prevalence of these rehospitalizations may, again, reflect opportunistic behavior of those evaluating reasons for readmission. On the other hand, it may be a symptom of high-quality care (during the period 2010–2015, there were no serious medical errors reported in the hospital that would result in damages awarded by the court). Organizational problems, namely diagnostic difficulties due to care for cost optimization (code 5a) and due to diagnostic unavailability (code 5b) together constituted 6% of all readmissions, suggesting that the hospital should place a greater focus on diagnostic issues. Readmissions associated with specificity of the reimbursement process to the third-party payer were rare (code 12—4%). However, in some sectors their magnitude was much higher. For example, in ophthalmology, they accounted for 29% of all cases and these readmissions could have been avoided if reimbursement rules were changed. The relatively high share of readmissions was due to the treatment pathways developed during index hospitalization (codes 1 and 2—8.6% in total in total) and only a few were due to patients’ noncompliance to therapeutic recommendations after index hospitalization (code 3—2.4%).

Most of the readmissions identified were related to the index hospitalization and among them the unplanned ones prevailed (63.9% of total) over the planned ones (26.5%). It seems that these categories of readmissions are natural candidates for reduction. However, not all are under provider control, e.g., reimbursement regulations or patients’ noncompliance. Less than one in ten rehospitalizations were unrelated to the index admission and these are possibly the ones that are the most difficult to tackle.

The multilevel logistic regression estimates show that being female, more than 35 km distance from patient’s home to hospital, longer than on average and very short LOS, higher ACCI score, and a larger number of admissions in a sector where the index hospitalization took place were all associated with higher odds of readmission. On the other hand, older age (50 and more years) and LOS lasting from 1.01 to 4 days were indicative for a lower risk of readmission.

According to our estimates, women were at higher risk of readmission and this finding was in contrast to other studies using single-center settings, where no significant effect of gender was identified [9,37,38,39]. Moreover, according to a systematic review on risk prediction models for readmission, the gender variable in most studies “did not contribute enough to be included in the final model” [6]. However, recent studies using multilevel modeling identified gender as a significant predictor of readmission risk, but they are not conclusive on which gender is at higher risk. Two American studies, each using data on 0.5 million admissions, identified males as more prone to readmission among Californian patients aged 50 or older [19] and Medicare beneficiaries in Texas [20]. On the other hand, women were at higher risk of being rehospitalized in two other American studies concerned with ischemic stroke Medicare beneficiaries [21] and patients after radical cystectomy for bladder cancer [28]. Considering age, our finding that older patients were at lower risk of readmission might be surprising because health deteriorates with increased age resulting in more potential problems that can cause readmission. However, similarly to our finding, some studies report a negative association between age and readmission risk [43]. Patients residing far from the hospital (>35 km) were at higher risk of readmission and this possibly reflects the fact that they experienced more difficulties in obtaining post-discharge out-patient care in clinics located in the analyzed hospital. Being treated by the same provider, or even the same physician, during hospitalization and in out-patient settings afterwards allows for the delivery of more comprehensive care and those residing far from the hospital experienced more difficulties in benefiting from such synergy. A longer than average stay during the index hospitalization was also associated with higher odds of readmission, and this result is in line with findings from studies using data from 11 Canadian hospitals located in Ontario [44], six American academic hospitals [45], and a state-wide study of readmission to a different hospital in California [19]. However, in our study, very short admissions (<1 days) were also prone to the highest risk of readmission. Comorbidity is another factor predisposing rehospitalization in our study and an ACCI score ≥2 was associated with ~10–11 times higher odds of readmission. This finding is in line with numerous other studies, which also show that higher values of Charlson score at the index hospitalization are indicative of future readmission [45,46].

In contrast to most other studies, we constructed a model that explains the variation in readmission risks to a satisfactory degree. The c-statistic value of 0.749 is higher than in several other studies on the topic [6]; however, the model cannot be considered as effective.

Several caveats apply to our analysis. (1) The study used data from one university hospital, and our results are possibly valid only for similar providers, thus, generalizing these findings to other types of hospitals may be risky. (2) In assessing the reasons for rehospitalization, we relied on the judgments of physicians who treated a readmitted patient, and these findings can reflect bias resulting from opportunistic behavior, as explained above. In addition, the assessment of readmission reasons by a single doctor was not validated by another reviewer, and it is unknown how the structure of readmission reasons would differ if such validation was applied. Therefore, the results regarding reasons for rehospitalization should be treated with special caution. Still, we believe that in this first attempt to assess the magnitude of readmissions in Poland, our analysis provides useful evidence for both hospitals and policymakers. In future studies, this limitation could be addressed to validate our findings. (3) We were able to retrieve limited data on patient characteristics and also factors other than those included in our analysis could play a role in the readmission risk. Particularly, no information on marital status, education, use of health care prior to the index hospitalization, and after discharge or self-rated health status was available in hospital electronic databases. Collecting data on these characteristics during hospitalization or merging hospital’s and third-party payer’s datasets could improve the process of predicting readmission risks. (5) We only identified rehospitalizations into the same hospital and, if a patient was readmitted elsewhere, such a case was not included in our analysis. This fact biased the RaR value downward. However, the approach based on single-unit data is accepted in the research on readmissions [9,37,38,39]. (6) We were not able to include data on any self-reported pre-admission performance-based measure of basic activities of daily living or even a measure of self-assessed health status as these were not recorded uniformly across all the clinics in the hospital. Furthermore, because the study included a wide range of medical specialties resulting in a great heterogeneity of patients, we could not include any consistent variable describing pharmacological treatment. This last limitation clearly results from the fact that we used retrospective data, which was already available in electronic patient records. Therefore, in the absence of such data in a hospital database, future studies of hospital readmissions in Poland should address this shortcoming by collecting more comprehensive information using a prospective study design.

## 5. Conclusions

Our study is the first to provide some evidence on the magnitude of hospital readmissions in Poland. Furthermore, because quality and efficiency issues have gained increasing importance in health policy, the topic should be further pursued to identify and implement evidence-based practices that would improve hospitals’ performance.

## Figures and Tables

**Table 1:** Selected characteristics of patients and hospital sectors.

	Hospitalizations (*N*)	Hospitalizations (%)	Readmissions (*N*)	Readmissions (%)	Readmissions Rate
Number of cases/Rate	34,008	-	3789	-	12.5
PATIENTS’ CHARACTERISTICS					
Gender			χ^2^ = 0.1, *p* = 0.716		
Male	16,492	48.5	1848	48.8	12.6
Female	17,516	51.5	1942	51.2	12.5
Age group, years			χ^2^ = 1346.1, *p* < 0.001		
0–4	3312	9.7	779	20.6	30.8
5–17	4292	12.6	954	25.2	28.6
18–29	1826	5.4	136	3.6	8.0
30–49	4379	12.9	344	9.1	8.5
50–65	8633	25.4	657	17.3	8.2
66 and more	11,566	34.0	919	24.3	8.6
Mean ± SD	48.9 ± 27.4		36.5 ± 30.0		
Place of residence, population			χ^2^ = 110.8, *p* < 0.001		
rural	10,448	30.7	1408	37.2	15.6
urban—up to 20.000	4834	14.2	504	13.3	11.6
urban—20.001–50.000	1190	3.5	136	3.6	12.9
urban—50.001–200.000	2226	6.5	289	7.6	14.9
urban—200.001–500.000	15,190	44.7	1438	38.0	10.5
urban—above 500.000	120	0.4	14	0.4	13.2
Distance from patient’s home to hospital, km *			χ^2^ = 197.3, *p* < 0.001		
up to 15	14,896	43.8	1373	36.2	10.2
16 to 35	4161	12.2	350	9.2	9.2
36 and more	14,920	43.9	2066	54.5	16.1
Type of admission			χ^2^ = 18.6, *p* < 0.001		
emergency	13,475	39.6	1379	36.4	11.4
scheduled	20,533	60.4	2410	63.6	13.3
Day of admission			χ^2^ = 9.0, *p* = 0.003		
week-day	29,323	86.2	3327	87.8	12.8
week-end	4685	13.8	462	12.2	10.9
Day of discharge from the hospital			χ^2^ = 6.0, *p* = 0.014		
week-day	30,476	89.6	3439	90.8	12.7
week-end	3532	10.4	350	9.2	11.0
Time of admission			χ^2^ = 3.0, *p* = 0.228		
7.00 a.m.–2.59 p.m.	27,175	79.9	2992	79.0	12.4
3.00 p.m.–10.59 p.m.	5627	16.5	664	17.5	13.4
11.00 p.m.–6.59 a.m.	1206	3.5	133	3.5	12.4
Length of stay, days			χ^2^ = 509.5, *p* < 0.001		
up to 1	3276	10.8	863	22.8	35.8
1.01 to 4	15,139	51.0	1428	37.7	10.4
4.01 to 7	4974	16.5	575	15.2	13.1
7.01 to 14	4058	13.4	564	14.9	16.1
14.01 and more	2772	9.2	359	9.5	14.9
Mean ± SD	6.2 ± 11.0		6.1 ± 10.9		
Age-adjusted Charlson comorbidity index, score			χ^2^ = 437.6, *p* < 0.001		
0–1	11,704	34.4	1182	31.2	11.2
2–3	9602	28.2	1598	42.2	20.0
4–5	9482	27.9	749	19.8	8.6
6 and more	3220	9.5	260	6.9	8.8
Mean ± SD	2.6 ± 2.2		2.3 ± 2.0		
HOSPITAL SECTORS’ CHARACTERISTICS					
Sectors by number of hospitalizations			χ^2^ = 541.9, *p* < 0.001		
up to 1000	4449	13.1	259	6.8	6.2
1001 to 3000	11,457	33.7	844	22.3	8.0
3001 and more	18,102	53.2	2686	70.9	17.4
Sectors by number of physicians			χ^2^ = 537.8, *p* < 0.001		
up to 1000	4973	14.6	310	8.2	6.6
1001 to 3000	10,933	32.1	793	20.9	7.8
3001 and more	18,102	53.2	2686	70.9	17.4
Sectors by number of cases			χ^2^ = 739.1, *p* < 0.001		
up to 1000	3661	10.8	117	3.1	3.3
1001 to 3000	15,131	44.5	1232	32.5	8.9
3001 and more	15,216	44.7	2440	64.4	19.1

Notes: * data not available for 31 hospitalizations.

**Table 2:** Readmissions numbers and rates in hospital sectors.

Sector	Hospitalizations	Readmissions	Readmissions Rate (%)	Share in Total Readmissions (%)
Pediatrics	6992	1771	33.9	46.7
Transplantation	788	153	24.1	4.0
Urology	2168	316	17.1	8.3
Vascular surgery	1004	106	11.8	2.8
Liver surgery	2153	179	9.1	4.7
Internal medicine	7470	619	9.0	16.3
Ophthalmology	3640	296	8.9	7.8
Psychiatry	693	50	7.8	1.3
Otolaryngology	1729	87	5.3	2.3
Dermatology	1439	66	4.8	1.7
Neurosurgery	1270	56	4.6	1.5
Strokes	943	29	3.2	0.8
Plastic surgery	625	17	2.8	0.4
Palliative medicine	88	2	2.3	0.1
Orthopedics	1694	34	2.0	0.9
Neurological rehabilitation	336	3	0.9	0.1
Neurology	685	5	0.7	0.1
Rehabilitation	61	0	0.0	0.0
Orthopedic rehabilitation	230	0	0.0	0.0

**Table 3:** Reasons for readmissions according to their status in terms of planning and relation to index hospitalization.

Code	Reason for Readmission	Readmission Category	Number of Cases (Share in Total Readmissions)
1	Index hospitalization was diagnostic	A	151 (4.0%)
2	Certain circumstances found during index hospitalization that prevented from performing procedure	A	175 (4.6%)
3	Patient’s non-adherence to therapeutic recommendations after index hospitalization	C	92 (2.4%)
4a	Patient discharged too early due to clinic’s organizational factors	C	37 (1.0%)
4b	Patient discharged too early due to too small number of beds in relation to needs	C	6 (0.2%)
4c	Patient discharged too early due to inadequate clinical assessment on discharge	C	16 (0.4%)
4d	Patient discharged on own request	C	13 (0.3%)
5a	Diagnostic difficulties during index hospitalization due to care for cost optimization	C	108 (2.9%)
5b	Diagnostic difficulties during index hospitalization due to inadequate availability of diagnostics	C	116 (3.1%)
5c	Incomplete diagnostics during index hospitalization due to incorrect initial diagnosis	C	2 (0.1%)
6	Postoperative complications	C	144 (3.8%)
7a	Non-optimal therapy during index hospitalization due to patient refusal of planned treatment	C	5 (0.1%)
7b	Non-optimal therapy during index hospitalization due to incorrect initial diagnosis	C	2 (0.1%)
8	Readmission resulting from specific nature of the disease and its routine treatment practice; unavoidable	C	1631 (43.0%)
9a	Readmission due to disease different to that of the index hospitalization – scheduled readmission	B	123 (3.2%)
9b	Readmission due to disease different to that of the index hospitalization – emergency readmission	D	238 (6.3%)
10	Disease progression, e.g. cancer progression	C	219 (5.8%)
11	Infection acquired in index hospitalization not detected on discharge	C	29 (0.8%)
12	Results from specificity of reimbursement process with third-party payer (National Health Fund)	A	150 (4.0%)
13	Ailments not confirmed during readmission	D	3 (0.1%)
14	Radiotherapy sessions and chemotherapy cycles	A	529 (14.0%)

Notes: readmission categories: A, planned and related to index hospitalization (*n* = 1005; RaR = 3.3%); B, planned and unrelated to index hospitalization (*n* = 123; RaR = 0.4%); C, unplanned and related to index hospitalization (*n* = 2420; RaR = 8.0%); and D, unplanned and unrelated to index hospitalization (*n* = 241; RaR = 0.8%).

**Table 4:** Logistic regression estimates for determinants of readmissions.

	Dependent Variable: Readmission During 30 Days after Index Hospitalization			
	Odds Ratio	St. Error	95% CI	*p*-value
Gender (ref.: Men)				
Women	1.14	0.04	1.06–1.23	0.001
Age, years (ref.: 18–29)				
0–4	0.84	0.16	0.58–1.21	0.342
5–17	0.81	0.15	0.56–1.16	0.252
30–49	1.05	0.14	0.82–1.35	0.694
50–65	0.23	0.03	0.18–0.30	<0.001
66 and more	0.24	0.03	0.18–0.32	<0.001
Distance from patient’s home to hospital, km * (ref.: up to 15)				
16 to 35	0.89	0.06	0.78–1.02	0.090
36 and more	1.17	0.05	1.08–1.27	<0.001
Length of stay, days (ref.: 4.01 to 7)				
up to 1	1.94	0.14	1.69–2.22	<0.001
1.01 to 4	0.79	0.05	0.71–0.89	<0.001
7.01 to 14	1.43	0.10	1.24–1.64	<0.001
14.01 and more	1.51	0.13	1.28–1.78	<0.001
Age-adjusted Charlson comorbidity index, score (ref.: 0–1)				
2–3	9.65	0.62	8.50–10.96	<0.001
4–5	9.07	0.87	7.52–10.94	<0.001
6 and more	11.38	1.33	8.68–13.64	<0.001
Sectors by number of hospitalizations (ref.: up to 1000)				
1001 to 3000	4.47	2.38	1.58–12.67	0.005
3001 and more	9.81	6.72	2.56–37.56	0.001
Constant term	0.01	0.00	0.00–0.01	<0.001
*c* statistic = 0.749; 95% CI: 0.740–757 Log likelihood: −9865.6; χ^2^ = 1772.8; *p* < 0.001 Observations: 33,977. Minimum (maximum) number of observations per sector: 61 (7468)				

Notes: CI—confidence interval.

## Supplementary Materials

The following are available online at https://www.mdpi.com/1660-4601/16/13/2339/s1, Table S1: Dataset.
